# Risk prediction of recurrent ischemic stroke based on Carotid Plaque-RADS: construction and validation of a nomogram model

**DOI:** 10.3389/fnagi.2025.1646916

**Published:** 2025-08-18

**Authors:** Miao Qiao, Ting Zhou, Rui Wang, Yanhui Jiang, Huitao Liang, Lingcui Meng

**Affiliations:** Department of Ultrasound Imaging, The Second Affiliated Hospital of Guangzhou University of Chinese Medicine, Guangzhou, China

**Keywords:** recurrent ischemic stroke, Carotid Plaque-RADS, nomogram prediction model, risk factors, carotid stenosis

## Abstract

**Background and purpose:**

Ischemic stroke (IS) is characterized by a high recurrence rate and more serious repercussions. Recently, the Carotid Plaque Reporting and Data System (Carotid Plaque-RADS) has been introduced to gauge and forecast the risk of cerebrovascular incidents. More studies are required to confirm its predictive power for recurrent ischemic stroke (RIS). We aimed to create a nomogram model that can evaluate the likelihood of RIS, with Carotid Plaque-RADS serving as a crucial instrument in this model.

**Methods:**

We carried out a retrospective review of 286 patients diagnosed with acute IS at the Second Affiliated Hospital of Guangzhou University of Chinese Medicine between January 2020 and January 2025. The study population consisted of two groups: the IS group (129 patients) and the RIS group (157 patients), depending on whether they experienced a recurrence of IS. Carotid ultrasound examination and clinical data were gathered and classified according to Carotid Plaque-RADS. Independent risk factors for the RIS were determined using multivariate logistic regression analyses. Subsequently, we developed a nomogram model to forecast RIS risk and evaluated its performance.

**Results:**

The RIS and IS groups showed significant differences in low-density lipoprotein (LDL), hypertension, atrial fibrillation, severe carotid stenosis, and Carotid Plaque-RADS categories. Multivariate logistic regression analysis identified LDL, hypertension, atrial fibrillation, severe carotid stenosis, and Carotid Plaque-RADS as independent risk factors for RIS. The nomogram model built using these risk factors demonstrated good calibration (H-L goodness-of-fit test *P* = 0.354). Internal and external validation demonstrated that the calibration curves were consistent with the original curves. The nomogram model combining Carotid Plaque-RADS and clinical features showed area under the curve (AUC) values of 0.79 and 0.76, outperforming models using only clinical features (AUC 0.72 and 0.70) or only Carotid Plaque-RADS (AUC 0.71 and 0.69). The model showed considerable clinical benefit within the 0.2–0.8 threshold range in the decision curve analysis (DCA).

**Conclusion:**

The nomogram model based on Carotid Plaque-RADS provides a novel and effective tool for clinical risk assessment and demonstrates favorable predictive performance for RIS.

## 1 Introduction

Ischemic stroke (IS) constitutes 65.3% of all stroke cases and stands as a primary contributor to mortality and disability on a global scale (GBD 2021 Stroke Risk Factor Collaborators, 2024; Thayabaranathan et al., 2022). Its high incidence and severe consequences pose a significant challenge to public health. A key pathogenic mechanism of IS is the detachment of unstable atherosclerotic plaques, which leads to thrombosis, arterial occlusion, and subsequent brain tissue ischemia and necrosis (Bazan et al., 2022). Research shows the 10-year cumulative recurrence rate of recurrent ischemic stroke (RIS) can reach 39.7% with a 10.4% first-year recurrence rate (Lin et al., 2021). RIS is linked to more severe sequelae such as higher cognitive impairment and long-term paralysis risks (Aini et al., 2023), making accurate risk assessment vital for RIS prevention.

The ESSEN Stroke Risk Score (ESSEN) primarily relies on demographic parameters like age and gender together with traditional risk factors (hypertension and diabetes), it fails to fully integrate plaque morphologic heterogeneity. A large cohort study showed the ESSEN score had an area under the curve (AUC) of 0.65 for predicting RIS, indicating 35% of high-risk individuals might be misclassified as low-risk (Huang et al., 2020) due to a limitation stemming from its inability to capture key pathologic events including intraplaque hemorrhage (IPH), fibrous cap (FC) rupture, and other pathological events reflecting dynamic evolution of plaque vulnerability that underlie thromboembolic events (Bazan et al., 2022; Brinjikji et al., 2016; Biswas et al., 2021).

Carotid ultrasound exhibits significant advantages in assessing vulnerable plaques in carotid arteries. It is widely used for its low cost, time efficiency and absence of radiation exposure (Brinjikji et al., 2016; Biswas et al., 2021). Carotid ultrasound can observe plaque characteristics and identify stenosis or occlusion by monitoring hemodynamic changes in the lumen. Studies have shown that vulnerable plaques are one of the major contributors to IS, and carotid ultrasound provides clinicians with important diagnostic information by assessing the location, size and morphology (Brinjikji et al., 2016; Zhao et al., 2017; Lyu et al., 2021; Yang et al., 2018). However, although carotid ultrasound is excellent in assessing the physical characteristics of plaques, it lacks clear evaluation criteria for plaque stability.

Carotid Plaque Reporting and Data System (Carotid Plaque-RADS) is a standardized categories that has recently been introduced by researchers from multiple countries (Saba et al., 2024). As a standardized carotid plaque categories system, Carotid Plaque-RADS performs morphological analysis of carotid plaques to deliver a comprehensive assessment of plaque stability. Building on morphologic risk features, the system assigns categories 1–4, with category 4 denoting progressively higher-risk plaques that harbor complex, vulnerable components. By integrating detailed plaque morphology [e.g., fibrous-cap integrity, lipid-rich necrotic core (LRNC), and intraplaque hemorrhage] with the severity of luminal stenosis. It provides morphological analysis of carotid plaque, which comprehensively assesses plaque stability, quantify the severity of atherosclerosis by integrating the morphology of plaque components with the degree of carotid stenosis, and provide a standardized description of carotid atherosclerotic lesions, providing a new perspective for stroke risk assessment. However, the utility of Carotid Plaque-RADS in predicting RIS compared with traditional diagnostic methods for IS requires further investigation. Therefore, this study employs Carotid Plaque-RADS to analyze carotid plaques and constructs a nomogram prediction model to assess its predictive potential for RIS.

## 2 Materials and methods

### 2.1 Study subjects

**FIGURE 1:**
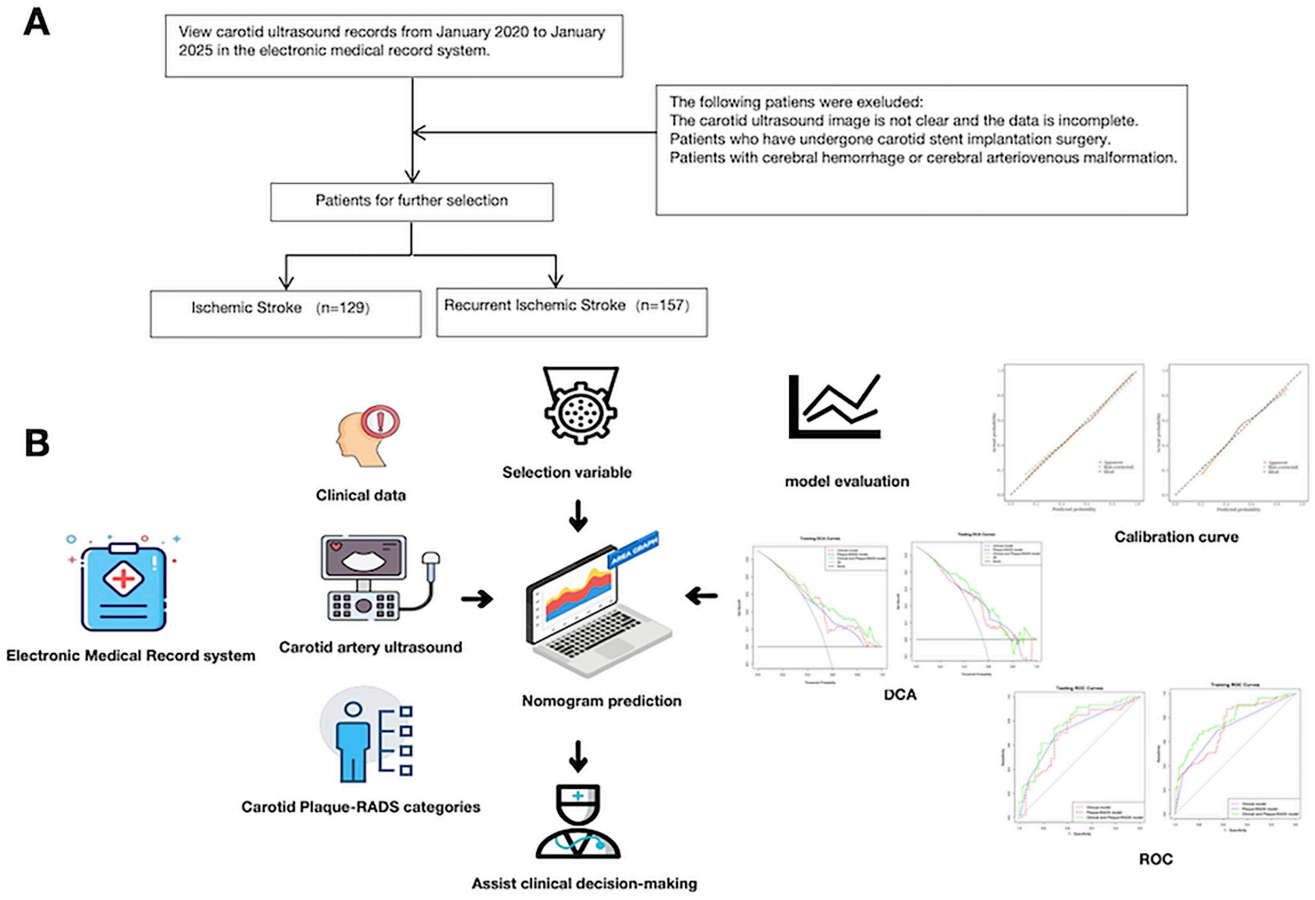
**(A)** The flowchart of all patients’ selection. **(B)** The conceptual framework of this study, including data collection, model development, and evaluation.

The study utilized the imaging examination database of the Second Affiliated Hospital of Guangzhou University of Chinese Medicine. We retrospectively analyzed carotid ultrasound examination data from January 2020 to January 2025 to identify eligible patients. All patients were inpatients in the neurology department, admitted for symptoms related to cerebrovascular and cardiovascular diseases. The study population consisted of two groups: the IS group and the RIS group. The IS group included patients who presented to our hospital for the first time with a stroke and no history of stroke. When included in the study, these patients did not experience a recurrence during follow-up. The RIS group included patients who had previously experienced an IS before undergoing carotid ultrasound examination and subsequently had a RIS during the follow-up period. Inclusion criteria: ① first-time diagnosis of IS at our hospital, supported by clinical symptoms and cranial CT or MRI findings; ② detection of carotid plaques with good-quality ultrasound images, complete laboratory tests and clinical data. Exclusion criteria included: ① poor image quality or incomplete data from carotid ultrasound examination; ② prior carotid stenting; ③ hemorrhagic stroke, cerebral hemorrhage, or cerebral artery malformation confirmed by head CT or MRI. Finally, 286 patients were enrolled in the study: the IS group (129 patients) and the RIS group (157 patients) (Figure 1). The Second Affiliated Hospital of Guangzhou University of Chinese Medicine ethics review board has approved this retrospective study (Ethical Approval No.: ZE2025-109-01). Written informed consent from patients was not required.

### 2.2 Clinical information

Clinical information was collected from the electronic medical record system. Including demographic data [gender, age, height, weight, and body mass index (BMI)], medical history [diabetes, hypertension, coronary heart disease, atrial fibrillation, and transient ischemic attack (TIA)], lifestyle factors (smoking status and drinking status), and laboratory indicators: total cholesterol (TC), high-density lipoprotein cholesterol (HDL), low-density lipoprotein cholesterol (LDL), triglycerides (TG), glycated hemoglobin (HbA1c), and fasting blood glucose (FBG).

Body mass index is calculated by dividing weight in kilograms by height in meters squared. Regarding lifestyle factors, drinking status was defined as an average ≥45 g daily for women and ≥90 g for men over the past decade. During that period, smoking status was defined as smoking at least of one cigarette daily. The following were the diagnostic standards for medical history: diabetes was defined as FBG ≥ 7.0 mmol/L or 2-h postprandial glucose levels ≥11.1 mmol/L. Hypertension was diagnosed based on systolic blood pressure ≥ 140 mmHg, diastolic blood pressure ≥ 90 mmHg, or the use of antihypertensive medication. Coronary heart disease was confirmed by imaging examinations, electrocardiograms, and typical symptoms such as chest pain or dyspnea on exertion (coronary angiography showing stenosis ≥50%). Atrial fibrillation was identified by electrocardiogram features. TIA was defined as a transient neurological deficit lasting less than 24 h without infarct lesions on imaging. Abnormal laboratory indicators were defined as follows: TG ≥ 1.7 mmol/L; LDL ≥ 3.4 mmol/L; HDL < 1.0 mmol/L (for men) or <1.3 mmol/L (for women); TC ≥ 5.2 mmol/L; HbA1c ≥ 6.5%; and FBG ≥ 7.0 mmol/L.

### 2.3 Carotid ultrasound examination

Carotid ultrasound examinations were performed using the Epic 7 ultrasound diagnostic device (4–8 MHz) and the IU Elite ultrasound diagnostic device (3–9 MHz). Plaque image analysis was conducted only on carotid arteries with patent lumens. When both carotid arteries were patent, the thickest plaque was selected for detailed examination. The location, size, and shape of the plaque were meticulously recorded, and the formula for calculating the degree of stenosis is as follows: Stenosis (%) = [(Diameter of the normal distal internal carotid artery − narrowest internal carotid artery diameter in the stenotic segment) / Diameter of the normal distal internal carotid artery] × 100%, according to North American Symptomatic Carotid Endarterectomy Trial (NASCET) (Barnett, 1991), carotid stenosis degree was classified as mild (<30%), moderate (30%–69%), and severe (70%–99%). The maximum wall thickness (MWT) was defined as the vertical distance from the lumen to the adventitia interface to the top of the plaque. The FC was identified as an arcuate, linear echogenicity structure between the plaque and the lumen. A thin FC was indicated if there was local echo disruption or incomplete visualization.

### 2.4 Carotid Plaque-RADS categories

To ensure the evaluative accuracy of the Carotid Plaque-RADS, patient identities were anonymized. Their carotid ultrasound images were randomly assigned to two ultrasound physicians with over 5 years of experience for evaluation. The evaluators were unware of each other’s assessment results and unable to know patients’ follow-up result. In cases of discrepancy, a third, more experienced senior physician was consulted to determine the final classification. Prior to image interpretation, the ultrasound physicians received standardized training, and regular assessments of scoring consistency were conducted. Carotid plaques are categorized according to the Carotid Plaque-RADS categories (Saba et al., 2024): Carotid Plaque-RADS 1 indicates a normal vascular wall; Carotid Plaque-RADS 2 indicates a plaque with MWT < 3 mm and with no complex plaque features; Carotid Plaque-RADS 3a indicates MWT ≥ 3 mm, with LRNC and a thick FC, excluding complex plaque features; Carotid Plaque-RADS 3b indicates MWT ≥ 3 mm, with LRNC and a thin but intact FC; Carotid Plaque-RADS 3c indicates plaque ulceration, where “ulceration” refers to a surface cavity, known as a “healed ulcer plaque.” Carotid Plaque-RADS 4 is not associated with MWT thickness, but is graded by the presence of one of the following: IPH (4a), FC rupture (4b), or luminal thrombus (4c). Note: Active ulceration with luminal thrombus is classified under Carotid Plaque-RADS 4c, while “healed ulcer plaque” (3c) refers to a residual cavity without active flow communication.

### 2.5 Endpoint assessment

This study systematically followed up with 286 patients who underwent carotid ultrasound examinations. The follow-up period extended from the date of admission to January 2025. Follow-up was conducted via medical record review and telephone interviews. For patients lost to follow-up, efforts were made to retrieve data through alternative means, such as contacting family members or reviewing available medical records to minimize data loss. The primary endpoint was symptomatic RIS: new-onset neurological deficit lasting ≥24 h and confirmed by DWI-MRI within 7 days of symptom onset, excluding hemorrhage, and all-cause mortality was the secondary endpoint. Patients were followed for up to 60 months (median follow-up: 38 months, IQR: 24–52 months), with censoring occurred at the first RIS, death, or January 2025, whichever came first. IS was diagnosed using the TOAST (Trial of Org 10172 in Acute Stroke Treatment classification) criteria (Adams et al., 1993). RIS was defined as the occurrence of new focal neurological deficits lasting more than 24 h, confirmed by diffusion-weighted magnetic resonance imaging showing new acute cerebral infarction lesions in the territory supplied by the carotid artery on the same side as the previously identified stroke. To ensure consistent diagnosis, the same imaging protocols and clinical criteria were applied throughout the study.

The diagnostic process for stroke recurrence was standardized, with two neurologists independently assessing clinical symptoms and imaging features. Disagreements were resolved by the clinical research team, ensuring the reliability and reproducibility of the data. All neurologists involved in the study underwent training to ensure consistent application of the TOAST criteria and DWI imaging interpretation. In cases of multiple RIS, only the first event was included in the statistical analysis to avoid bias. The diagnostic process adhered to internationally accepted stroke classification and imaging standards to ensure the scientific validity and reproducibility of data collection.

### 2.6 Statistical analysis

Statistical analysis was performed using SPSS Statistics (version 25.0, IBM) and R (version 4.2.3). Continuous data were presented as mean ± standard deviation and assessed via *t*-test. Categorical data were expressed as percentages and assessed using Chi-square tests. Risk factors for stroke recurrence were determined by univariate and multivariate logistic regression analysis, adjusting for age, sex, smoking status, diabetes, and BMI as potential confounders. A nomogram prediction model was constructed, incorporating factors that were statistically significant in the multivariate analysis, to predict stroke recurrence. The clinical effectiveness of the nomogram prediction model was assessed via calibration curves and decision curve analysis. The model’s fit was evaluated using the Hosmer–Lemeshow test, and its predictive capability was measured by the receiver operating characteristic (ROC) curve, with the AUC calculated. A significance level of *P* < 0.05 was used throughout the analysis.

## 3 Result

### 3.1 Baseline characteristics

This study included 286 patients, comprising 194 males (68%) and 92 females (32%). Their ages ranged from 60 to 92 years, with a mean age of 73.36 years. Carotid plaques were detected in 256 patients (89%) within the entire study cohort. During a 60-month follow-up period ending in January 2025, 129 patients experienced an IS, while 157 experienced a RIS. Among the RIS group, 147 patients (93.6%) had carotid plaques detected, and 30 patients (20.4%) of them had a severe carotid stenosis. The distribution of Carotid Plaque-RADS categories was as follows: 10 patients (6.3%) in Carotid Plaque-RADS 1, 38 patients (24.2%) in Carotid Plaque-RADS 2, 58 patients (36.9%) in Carotid Plaque-RADS 3, and 51 patients (32.5%) in Carotid Plaque-RADS 4. In contrast, among the IS group, 109 patients (84.5%) had carotid plaques detected, and 8 patients (7.3%) of them had a carotid stenosis degree of ≥70%. The distribution of Carotid Plaque-RADS categories was as follows: 20 patients (15.5%) in Carotid Plaque-RADS 1, 65 patients (50.4%) in Carotid Plaque-RADS 2, 35 patients (27.1%) in Carotid Plaque-RADS 3, and 9 patients (7.0%) in Carotid Plaque-RADS 4. Further analysis revealed that the detection rate of carotid plaques and the degree of carotid stenosis were closely associated with the occurrence of stroke events, particularly in the RIS group, where the prevalence of plaques and severe stenosis was higher.

A total of 286 patients were randomly assigned in a ratio of 3:1 to create a training set of 200 patients and a validation set of 86 patients. The external validation cohort consisted of 86 patients retrospectively enrolled from the same single-center temporal validation cohort (2019–2024) with identical inclusion criteria, representing a temporally distinct but demographically similar population. Within the training set, patients were further divided into two groups: the IS group, comprising 92 cases, and the RIS group, comprising 108 cases. Patients were divided according to whether they had a stroke recurrence during the follow-up period. We compared the training and validation sets with respect to age, height, weight, BMI, TC, HDL, LDL, TG, HbA1c, FBG, gender, smoking status, drinking status, hypertension, diabetes, TIA, coronary heart disease, atrial fibrillation, fibrinogen, carotid stenosis degree, and Carotid Plaque-RADS categories. There was no significant difference between the two groups (*P* > 0.05), indicating that comparisons can be made (Table 1).

**TABLE 1:** Comparison of clinical and ultrasound data between training and validation sets.

Characteristic	Training set (*n* = 200)	Validation set (*n* = 86)	*t*/χ^2^	*P*
Age	69.04 ± 10.65	70.10 ± 9.79	0.79	0.428
Height	162.75 ± 7.92	163.50 ± 7.29	0.75	0.453
Weight	62.33 ± 12.03	63.10 ± 10.50	0.52	0.602
BMI	23.43 ± 3.73	23.56 ± 3.41	0.28	0.779
TC	4.23 ± 1.16	4.02 ± 1.32	1.32	0.187
HDL	2.07 ± 12.03	1.06 ± 0.29	−0.78	0.439
LDL	2.64 ± 0.84	2.53 ± 1.02	−0.91	0.362
TG	1.54 ± 1.11	1.72 ± 1.43	1.16	0.246
HbA1c	6.94 ± 4.11	6.97 ± 1.93	0.07	0.942
FBG	7.53 ± 4.33	7.52 ± 3.25	−0.02	0.985
Fibrinogen	3.94 ± 2.52	3.95 ± 1.45	0.01	0.993
Gender, *n* (%)			0.03	0.854
Male	135 (67.50)	59 (68.60)		
Female	65 (32.50)	27 (31.40)		
Smoking status, *n* (%)			0.80	0.37
No	131 (65.50)	61 (70.93)		
Yes	69 (34.50)	25 (29.07)		
Drinking status, *n* (%)			0.21	0.648
No	176 (88.00)	74 (86.05)		
Yes	24 (12.00)	12 (13.95)		
Hypertension, *n* (%)			0.62	0.431
No	53 (26.50)	19 (22.09)		
Yes	147 (73.50)	67 (77.91)		
Diabetes, *n* (%)			0.02	0.899
No	124 (62.00)	54 (62.79)		
Yes	76 (38.00)	32 (37.21)		
TIA, *n* (%)			0.45	0.503
No	186 (93.00)	78 (90.70)		
Yes	14 (7.00)	8 (9.30)		
Coronary heart disease, *n* (%)			3.5	0.061
No	184 (92.00)	75 (87.21)		
Yes	16 (8.00)	11 (12.79)		
Atrial fibrillation, *n* (%)			0.06	0.801
No	181 (90.50)	77 (89.53)		
Yes	19 (9.50)	9 (10.47)		
Carotid stenosis degree, *n* (%)			4.48	0.058
Mild–moderate	182 (91.00)	72 (83.72)		
Severe	18 (9.00)	14 (16.28)		
Carotid Plaque-RADS (%)			6.54	0.088
Carotid Plaque-RADS 1	25 (12.50)	5 (5.81)		
Carotid Plaque-RADS 2	77 (38.50)	26 (30.23)		
Carotid Plaque-RADS 3	58 (29.00)	35 (40.70)		
Carotid Plaque-RADS 4	40 (20.00)	20 (23.26)		

### 3.2 Univariate analysis of associations between the two groups

In the univariate analysis, the RIS group exhibited significantly higher levels of LDL compared to the IS group (*P* < 0.05). No statistically significant differences were found between the two groups in height, weight, BMI, TC, TG, HDL, HbA1c, FBG, or fibrinogen levels (*P* > 0.05; Table 2). Significant differences were observed between the groups for hypertension, atrial fibrillation, carotid stenosis degree, and Carotid Plaque-RADS categories (*P* < 0.05), while no significant differences were found for gender, smoking status, drinking status, TIA, diabetes, or coronary heart disease (*P* > 0.05; Table 2). Additionally, the Carotid Plaque-RADS categories showed significant differences between the RIS and IS groups (*P* < 0.05; Table 2).

**TABLE 2:** Comparison of data between recurrent ischemic stroke group and first stroke group.

Characteristic	The RIS group (*n* = 108)	The IS group (*n* = 92)	*t*/χ^2^	*P*
Age	68.55 ± 10.61	69.24 ± 10.97	0.45	0.651
Height	163.73 ± 7.15	162.08 ± 8.69	−1.48	0.141
Weight	63.53 ± 10.79	61.48 ± 12.95	−1.22	0.224
BMI	23.62 ± 3.30	23.29 ± 4.05	−0.64	0.524
TC	4.13 ± 1.08	4.29 ± 1.25	0.96	0.34
HDL	2.67 ± 16.35	1.19 ± 0.64	−0.87	0.387
LDL	2.73 ± 0.79	2.49 ± 0.97	−1.89	0.06
TG	1.73 ± 1.53	1.65 ± 1.20	−0.41	0.682
HbA1c	7.40 ± 5.37	6.79 ± 1.95	−1.03	0.304
FBG	7.65 ± 3.72	7.83 ± 5.03	0.30	0.767
Fibrinogen	4.11 ± 3.25	3.74 ± 1.13	−1.06	0.293
Gender, *n* (%)			1.54	0.214
Male	77 (71.30)	58 (63.04)		
Female	31 (28.70)	34 (36.96)		
Smoking status, *n* (%)			0.00	0.976
No	73 (67.59)	62 (67.39)		
Yes	35 (32.41)	30 (32.61)		
Drinking status, *n* (%)			1.56	0.212
No	91 (84.26)	83 (90.22)		
Yes	17 (15.74)	9 (9.78)		
Hypertension, *n* (%)			11.66	<0.001
No	18 (16.67)	35 (38.04)		
Yes	90 (83.33)	57 (61.96)		
Diabetes, *n* (%)			0.00	0.972
No	66 (61.11)	56 (60.87)		
Yes	42 (38.89)	36 (39.13)		
TIA, *n* (%)			3.78	0.052
No	95 (87.96)	88 (95.65)		
Yes	13 (12.04)	4 (4.35)		
Coronary heart disease, *n* (%)			1.05	0.306
No	98 (90.74)	87 (94.57)		
Yes	10 (9.26)	5 (5.43)		
Atrial fibrillation, *n* (%)			7.71	0.005
No	92 (85.19)	89 (96.74)		
Yes	16 (14.81)	3 (3.26)		
Carotid stenosis degree, *n* (%)			9.61	0.002
Mild–moderate	84 (77.78)	86 (93.48)		
Severe	24 (22.22)	6 (6.52)		
Carotid Plaque-RADS (%)			30.68	<0.001
Carotid Plaque-RADS 1	8 (7.41)	12 (13.04)		
Carotid Plaque-RADS 2	27 (25.00)	49 (53.26)		
Carotid Plaque-RADS 3	33 (30.56)	24 (26.09)		
Carotid Plaque-RADS 4	40 (37.04)	7 (7.61)		

### 3.3 Multivariate binary logistic regression analysis between the two groups

Multivariate logistic regression analysis revealed that statistically significant variables in univariate analysis—LDL, hypertension, atrial fibrillation, severe carotid stenosis, and Carotid Plaque-RADS 2, 3, and 4—were all independent risk factors for RIS (*P* < 0.05; Table 3). Notably, while TIA was associated with recurrence in the univariate analysis (*P* < 0.05), its effect was no longer independently significant after adjusting for confounding variables in the multivariate logistic regression model (β = 1.02, *P* = 0.16). This suggests that the association between TIA and recurrence may be mediated by other risk factors or due to collinearity. Consequently, TIA was excluded from the final multivariate logistic regression model.

**TABLE 3:** Multivariate logistic regression analysis for predicting RIS.

Variables	β	SE	Wald	OR (95% CI)	*P*
LDL	0.34	0.16	4.45	1.40 (1.03 ∼ 1.92)	0.035
Carotid stenosis (severe)	0.37	0.44	10.43	1.45 (0.56 ∼ 3.76)	0.021
Hypertension	1.18	0.33	13.18	3.27 (1.73 ∼ 6.20)	<0.001
Atrial fibrillation	1.82	0.56	10.56	6.18 (2.06 ∼ 18.52)	0.001
Carotid Plaque-RADS 2	0.45	0.49	0.85	1.57 (0.60 ∼ 2.14)	0.044
Carotid Plaque-RADS 3	1.36	0.5	7.40	3.91 (1.46 ∼ 10.47)	0.007
Carotid Plaque-RADS 4	2.64	0.61	18.58	14.03 (4.22 ∼ 46.64)	<0.001

### 3.4 Construction of the RIS nomogram prediction model

**FIGURE 2:**
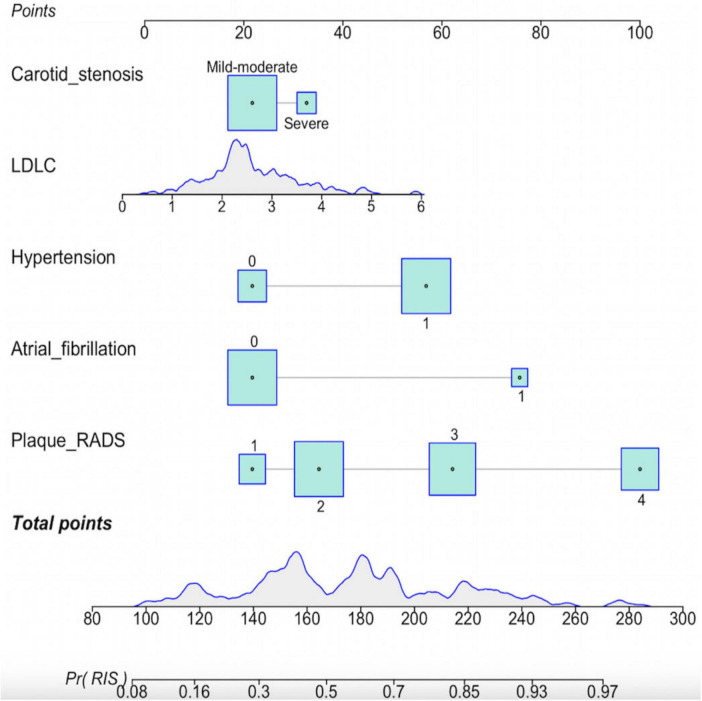
Nomogram for predicting the risk of RIS.

To facilitate the intuitive presentation of the predictive model, we developed a nomogram incorporating five risk factors (Figure 2). The total score is calculated by adding up the individual scores assigned to each risk factor. This score is used to estimate the risk of RIS. For example, a patient with IS who also has hypertension (42 points), severe carotid stenosis (12 points), Carotid Plaque-RADS categories of 4 (100 points), an LDL level of ≥6 mmol/L (78 points), and a history of atrial fibrillation (68 points) would accumulate a total score of 300 points. This score corresponds to a predicted RIS risk of over 90%, indicating an extremely high risk of recurrence.

### 3.5 Validation of the RIS nomogram prediction model

**FIGURE 3:**
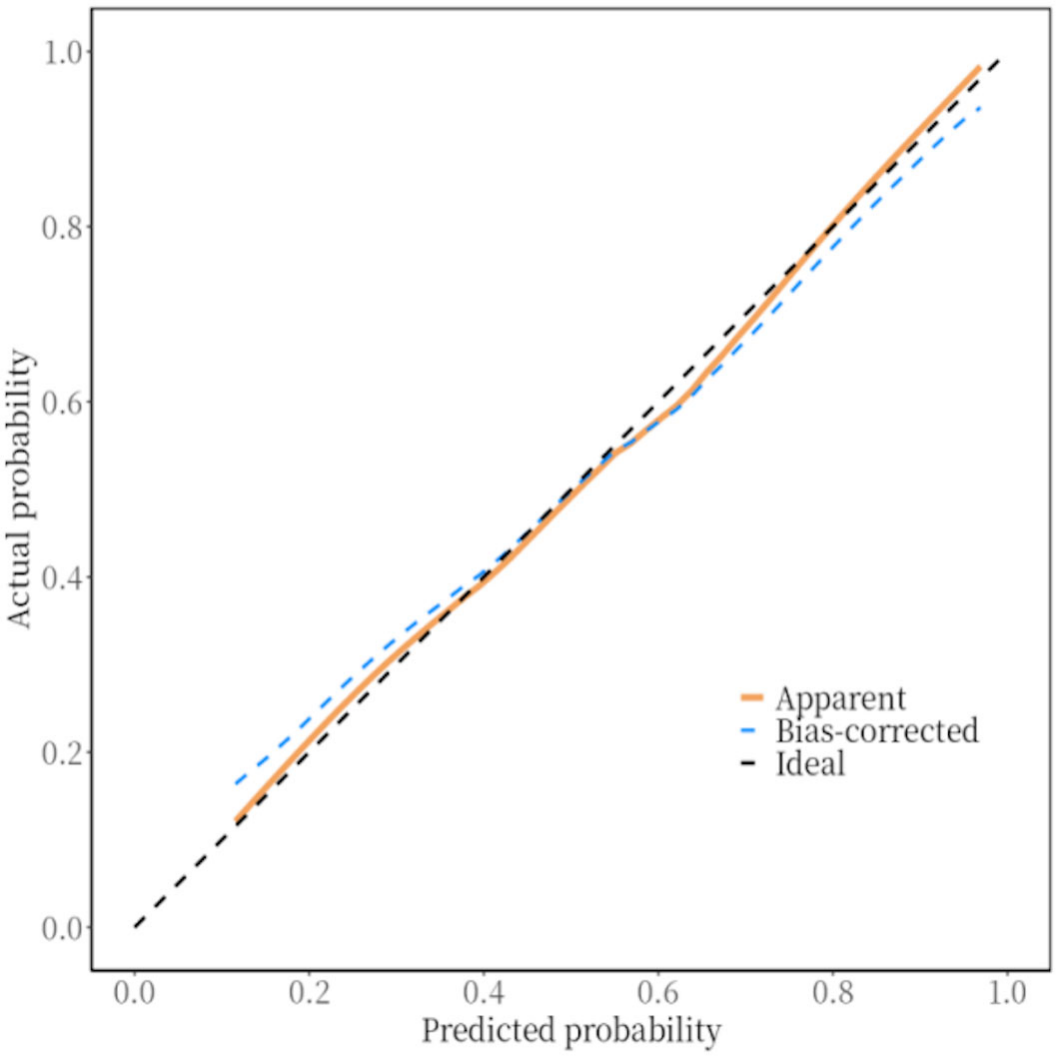
Internal validation calibration curve for the RIS nomogram prediction model.

**FIGURE 4:**
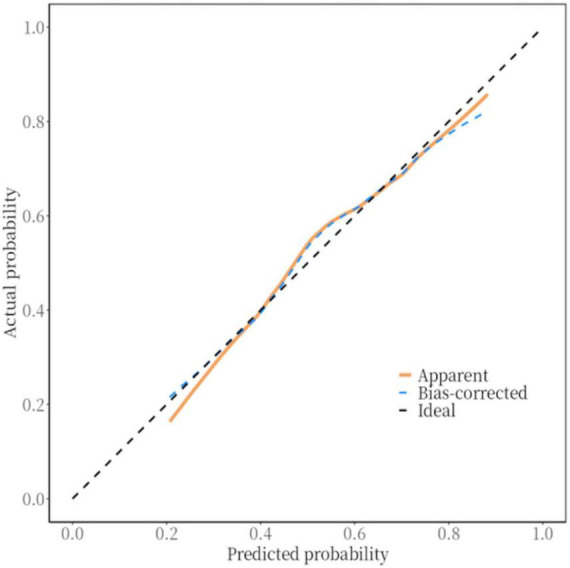
External validation calibration curve for the RIS nomogram prediction model.

**FIGURE 5:**
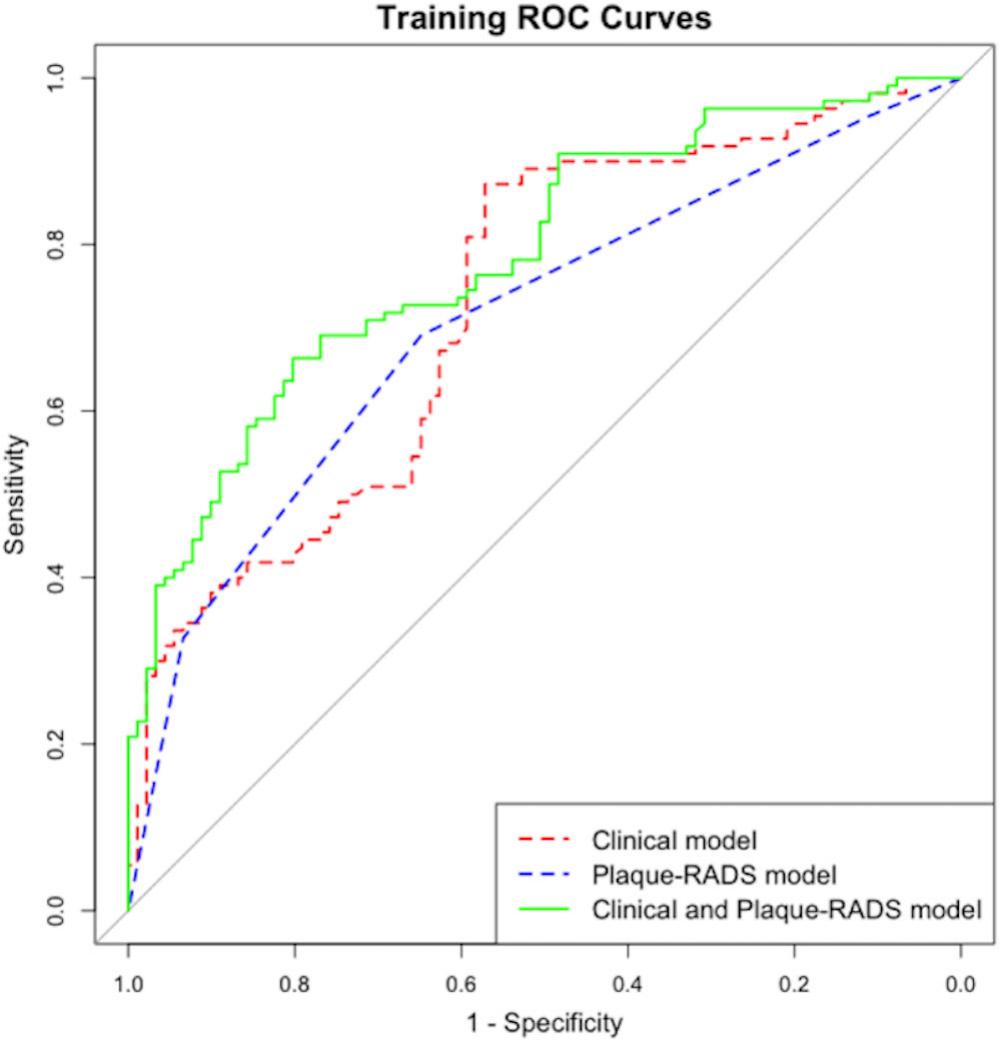
Internal validation ROC for the RIS nomogram prediction model.

**FIGURE 6:**
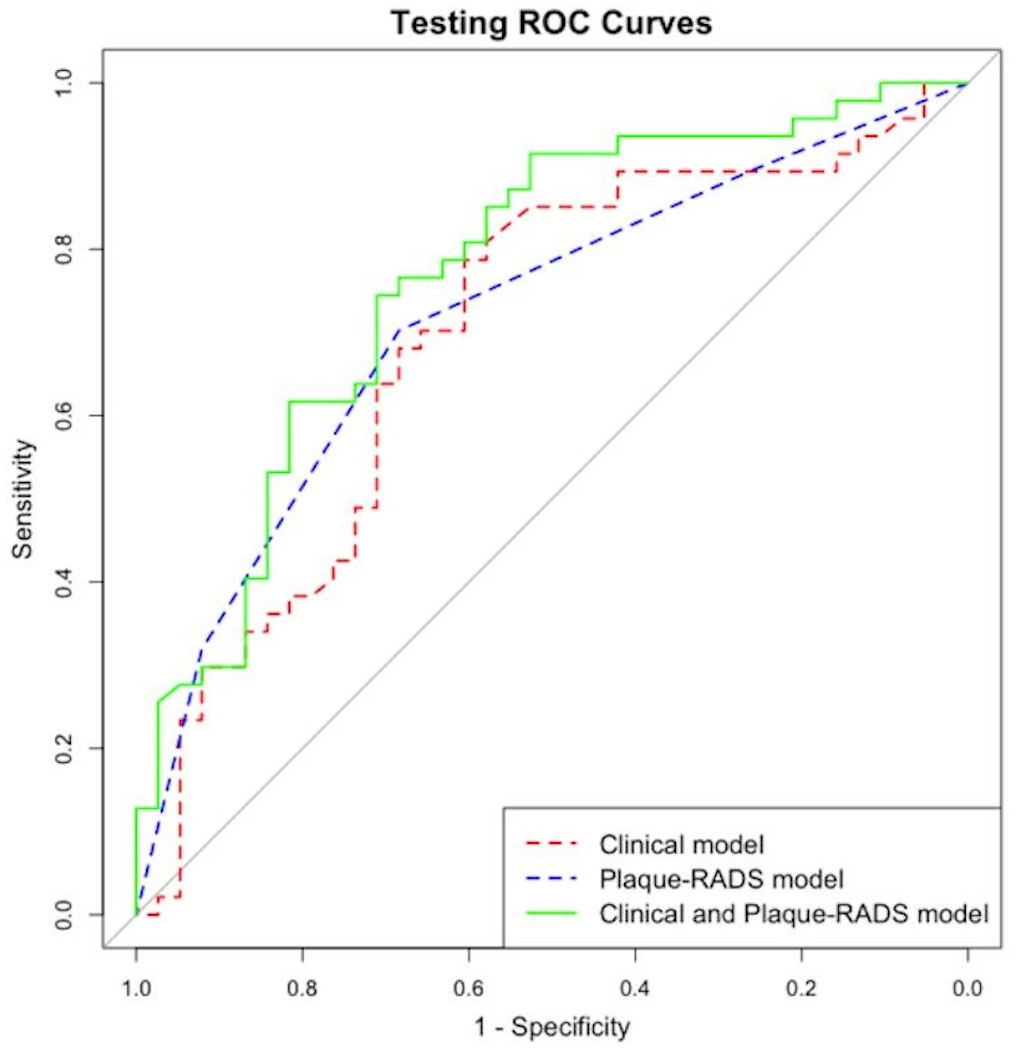
External validation ROC for the RIS nomogram prediction model.

The *P*-value of the Hosmer–Lemeshow test was 0.354, indicating a satisfactory fit. Internal and external for calibration curves validation are presented in Figures 3, 4, respectively, and demonstrate that the nomogram predictions for RIS are consistent with actual outcomes. The internal validation results demonstrated that the calibration curve nearly aligned with the ideal curve (Figure 3), suggesting that the model have strong calibration and discrimination abilities within the training set. Specifically, the combined model incorporating clinical features and Carotid Plaque-RADS achieved an AUC of 0.79 (95% CI: 0.76–0.81). In contrast the model using only clinical features had an AUC of 0.72 (95% CI: 0.69–0.75), and the model using only Carotid Plaque-RADS had an AUC of 0.71 (95% CI: 0.68–0.76) (Figure 5). External validation results showed the calibration curve aligned with the ideal curve (Figure 4), further confirming the model’s stability and applicability in an independent validation set. The combined model integrating clinical features and Carotid Plaque-RADS achieved an AUC of 0.76 (95% CI: 0.69–0.82). The models using only clinical features and only Carotid Plaque-RADS had AUCs of 0.70 (95% CI: 0.62–0.78) and 0.69 (95% CI: 0.60–0.79), respectively (Figure 6).

Consistency and stability analyses show the model demonstrate good predictive performance across diverse datasets, indicating substantial clinical application value. Integration of Carotid Plaque-RADS categories into clinical features significantly improves the model’s predictive accuracy (*P* < 0.05), enabling more precise identification of high-risk patients.

### 3.6 Decision curve analysis of the RIS risk nomogram prediction model

**FIGURE 7:**
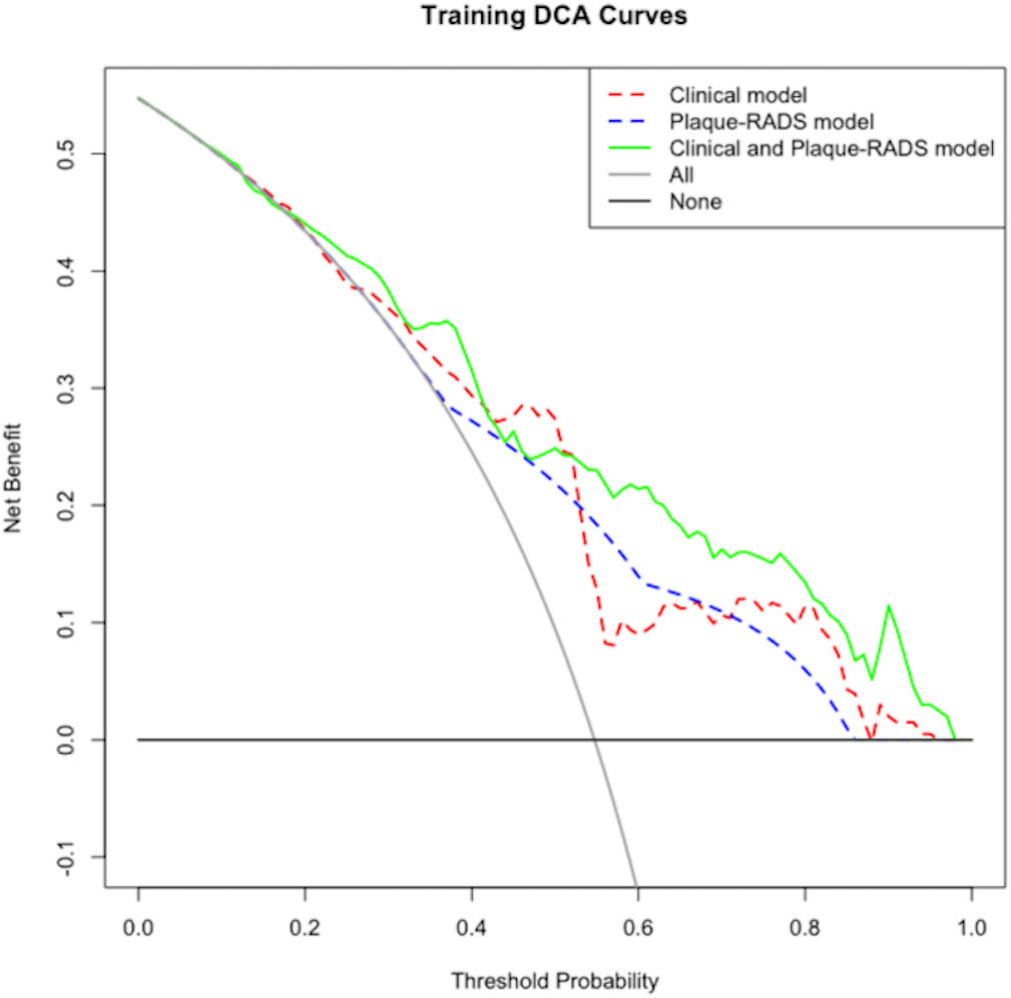
Internal validation DCA for the RIS risk nomogram prediction model.

**FIGURE 8:**
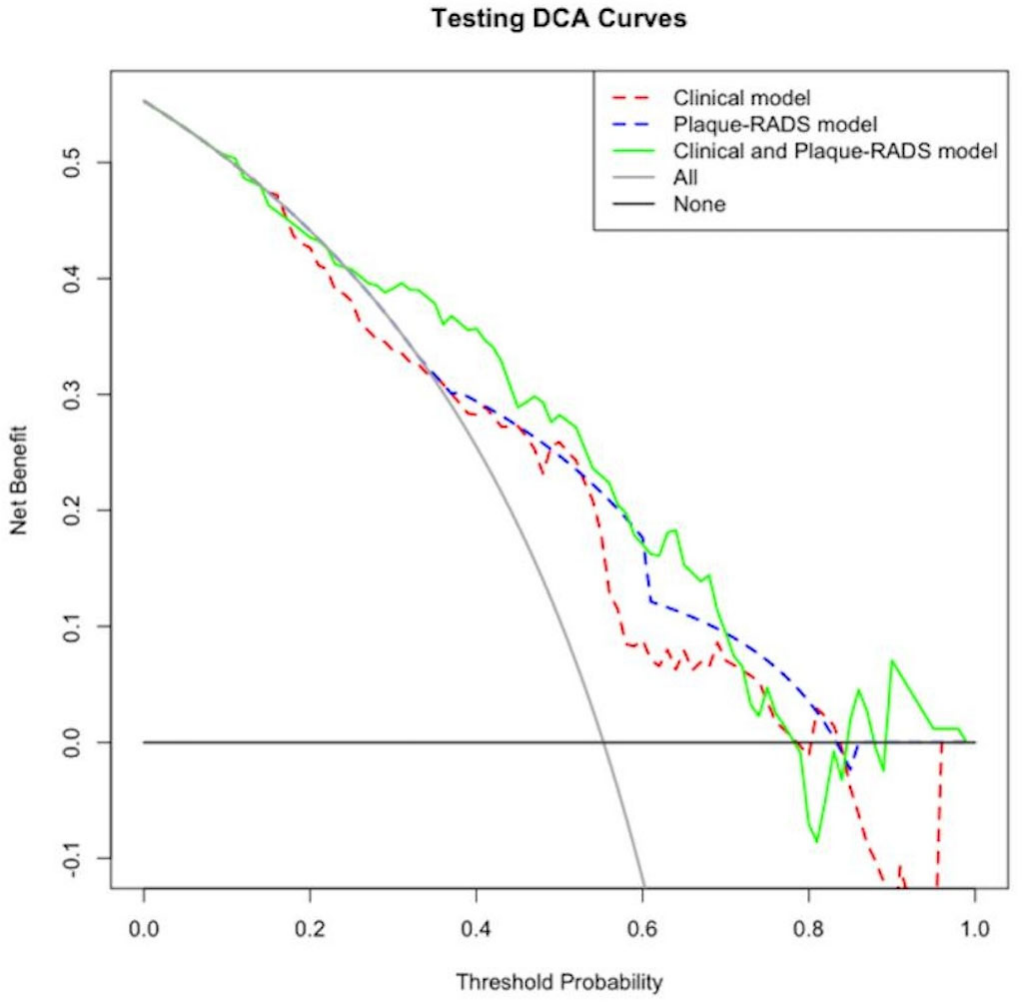
External validation DCA for the RIS risk nomogram prediction model.

Figures 7, 8 show decision curve analysis (DCA) curves applied to the RIS risk nomogram prediction model for the training and testing datasets respectively. The DCA curves for both datasets indicate the model provides substantial standard clinical net benefit and retains stable clinical utility advantages within the threshold probability range of 0.2–0.8. These results suggest the model has practical utility for clinical decision-making in RIS risk management.

## 4 Discussion

Accurate RIS risk assessment is critical for IS patients. The purpose of this study was to develop and validate a nomogram prediction model for RIS risk using Carotid Plaque-RADS. The nomogram model incorporating Carotid Plaque-RADS showed high diagnostic efficiency in both training and testing datasets with an AUC of 0.76, outperforming models using only clinical features (AUC 0.70) or only Carotid Plaque-RADS (AUC 0.69). Calibration curves closely matched ideal curves, confirming the model’s consistency and stability in an independent validation set. The model exhibited robust predictive performance across diverse datasets, indicating substantial clinical application value. The DCA curves further confirmed the model’s high accuracy and significant clinical net benefit. These results highlight the utility of integrating Carotid Plaque-RADS with clinical features to significantly enhance diagnostic performance and offer a new perspective for clinical risk stratification.

Previous studies have confirmed associations between hypertension, high LDL levels, atrial fibrillation, severe carotid stenosis and RIS (Kolmos et al., 2021; Yaghi et al., 2021; Lee et al., 2022). Our results align with these prior findings. As a systemic condition, atherosclerosis is influenced by multiple risk factors: hypertension, through vascular endothelial damage and reduced vascular elasticity, elevates plaque irregularity and thrombosis risks, establishing that it is one of the important independent risk factors for both incident and RIS (Howard et al., 2025). Concurrently, changes in plaque composition also elevate stroke risk (Kondakov and Lelyuk, 2021). Elevated LDL levels directly facilitate the formation and progression of atherosclerotic plaques. The deposition of LDL in the vascular endothelium triggers an inflammatory response through oxidation, which increases plaque instability and rupture risk, leading to thrombosis and RIS (Rajan et al., 2024; Peng et al., 2023). Atrial fibrillation also increases the risk of RIS via clot formation in the atria and subsequent embolism, particularly when anticoagulant therapy is not standardized, resulting in a significantly higher risk (Reddy et al., 2017). Severe carotid stenosis is a high-risk factor for stroke. Obstructed blood flow increases the risk of brain tissue hypoxia, making plaques more prone to rupture and bleeding, which further exacerbates vascular stenosis and increases the risk of RIS. These findings demonstrate that RIS results from the long-term combined effects of multiple risk factors. Enhancing secondary prevention strategies is crucial for reducing cardiovascular events.

Conventional stroke risk assessment systems depends on the degree of carotid stenosis to determine risk levels (Jonas et al., 2014), a technique widely adopted in clinical practice. This method has limitations as it does not fully account for diverse plaque morphological features and may overlook critical risk factors. Our study included 129 IS patients, of whom only 8 patients (7.3%) had severe carotid stenosis; among 157 RIS stroke patients, 30 patients (20.4%) had severe carotid stenosis. These results suggest that exclusive reliance on carotid stenosis for stroke risk assessment may underestimate risk of RIS in specific patient groups. Our study highlights the need to incorporate factors beyond traditional evaluations of luminal stenosis, particularly carotid plaque vulnerability assessment. Key plaque vulnerability indicators—IPH, FC rupture, and luminal thrombus—are critical for RIS risk prediction. Comprehensive evaluation of these features can improve RIS risk prediction accuracy and refine risk stratification.

Carotid Plaque-RADS as a standardized category provides a more definitive description of the morphological characteristics of carotid plaque. Recent studies have shown that assessing carotid plaque vulnerability characteristics can improve the accuracy of stroke and cardiovascular event prediction (Willeit et al., 2020), and serves as an independent stroke predictor. Our study further explored the association between Carotid Plaque-RADS and RIS by comparing Carotid Plaque-RADS and the degree of carotid stenosis among patients with RIS and IS who underwent Carotid Plaque-RADS evaluation, and found that the RIS group had a higher proportion of patients with Carotid Plaque-RADS 3 and Carotid Plaque-RADS 4 (69.4%) compared to the IS group (34.1%). Further analysis showed the combined effect of severe carotid stenosis (≥70%) and high Carotid Plaque-RADS grade (above 3 grade) were more pronounced in the RIS group, suggesting a synergistic influence on increasing stroke recurrence risk. Comprehensive analysis revealed that high Carotid Plaque-RADS grade and severe carotid stenosis are significant risk factors for predicting stroke recurrence. In addition, there was a significant correlation between the degree of carotid stenosis and Carotid Plaque-RADS in the RIS group compared with the IS group, and the risk of RIS increased with higher Carotid Plaque-RADS grade. This finding aligns with the study by Huang et al. (2025) and Howard et al. (2025), indicating that Carotid Plaque-RADS outperforms traditional stenosis assessment in stroke risk stratification, particularly in cases of mild to moderate stenosis. Significantly, plaques exhibiting mild to moderate carotid stenosis are capable of inducing cardiovascular events, while severe carotid atherosclerotic lesions might persist without symptoms over the long term (Lyu et al., 2021; Kondakov and Lelyuk, 2021; Rajan et al., 2024).

In our study, the risk factor with the greatest contribution in the nomogram prediction model is Carotid Plaque-RADS 4, with a score of 100 points. This is not hard to understand, Carotid Plaque-RADS classifies carotid plaques into different risk levels, among which high-risk plaques include 4a (IPH), 4b (FC rupture), and 4c (lumen thrombus). Mcnally et al. (2015) demonstrated that lumen thrombus is the most influential factor for carotid source stroke (OR value of 103.6), our study is consistent with previous findings. Therefore, in the Carotid Plaque-RADS system, lumen thrombus is classified as the highest category of 4c. The Carotid Plaque-RADS guidelines also mention auxiliary features such as plaque inflammation, neovascularization, and plaque calcification. These characteristics do not directly determine the main score of Carotid Plaque-RADS but can serve as supplementary information for further assessing the vulnerability and risk of plaques. However, due to limitations and unclear diagnostic criteria for some features, this study did not include the assessment of these auxiliary characteristics.

This study has several strengths. First, it innovatively integrates Carotid Plaque-RADS with traditional risk factors to develop a nomogram model for predicting RIS. This integration enriches the model’s predictive dimensions and enhances its performance. Second, the nomogram simplifies the complex prediction process into an intuitive visual tool, making it more practical for clinical use and aiding clinicians in quickly assessing patient risk and making informed treatment decisions. Third, we used clinical decision curves to evaluate the model’s clinical decision-making capabilities, in addition to the AUC. While the AUC measures the model’s predictive accuracy, clinical decision curves assess the net benefit to patients at different predictive probabilities, helping doctors make better-informed decisions.

This research has several limitations. Firstly, the model excluded important risk factors such as smoking status, BMI, diabetes, and TIA which were excluded from the multivariate model because they did not reach *P* < 0.05 in univariate analysis and failed to improve the AUC. During the data analysis, we applied various regression methods, which produced slightly different results. These differences likely stem from the retrospective study design and a restricted sample size. Secondly, relying exclusively on data from our hospital could result in selection bias and restrict the model’s applicability to more diverse populations. This limits the applicability of the model to more diverse populations, regions, and healthcare environments. Additionally, while Carotid Plaque-RADS 4 includes high-risk subcategories (4a: IPH, 4b: FC rupture, and 4c: luminal thrombus), limited sample size of Plaque-RADS 4 cases and individual subtypes precluded separate analyses. Future multicenter studies with larger cohorts are needed to validate the differential predictive value of these subcategories. Furthermore, the study relied solely on carotid ultrasound, which may underestimate IPH or FC rupture compared to MRI/CT angiography; future studies should validate Carotid Plaque-RADS using multimodal imaging. Multicenter prospective studies with larger, more diverse cohorts and multimodal imaging are required to validate Carotid Plaque-RADS subcategories and enhance the model’s generalizability.

## 5 Conclusion

In this investigation, we retrospectively gathered clinical and carotid ultrasound data from patients with IS, categorized them using Carotid Plaque-RADS, and pinpointed risk factors for RIS. On this basis, we devised a nomogram model to forecast the risk of RIS utilizing the Carotid Plaque-RADS categories. Subjected to both internal and external validation, the model exhibited robust discrimination, calibration, and predictive capabilities, surpassing a nomogram model reliant solely on clinical attributes. This model is capable of identifying patients at elevated risk of RIS and steering the formulation of tailored diagnostic and therapeutic strategies. The newly developed nomogram model plays a crucial role in bolstering communication between patients and healthcare providers and refining clinical decision-making processes. By identifying high-risk patients (nomogram score > 200), clinicians can intensify secondary prevention—for example, initiating dual antiplatelet therapy and escalate statin dosing to reduce actual RIS incidence and improve long-term functional outcomes. Follow-up research will focus on refining the classification criteria and conducting multi-center trials with larger sample sizes to further verify its clinical efficacy. Additionally, we plan to investigate the efficacy of interventions based on the Carotid Plaque-RADS categories in reducing the risk of stroke recurrence, thereby providing stronger support for clinical prevention and treatment strategies.

## Data Availability

The raw data supporting the conclusions of this article will be made available by the authors, without undue reservation.
